# Impact of physical activity on course and outcome of pregnancy from pre- to postnatal

**DOI:** 10.1038/s41430-021-00904-7

**Published:** 2021-04-07

**Authors:** Nina Ferrari, Christine Joisten

**Affiliations:** 1 Cologne Centre for Prevention in Childhood and Youth/ Heart Centre Cologne, University Hospital of Cologne, Cologne, Germany; 2 Department for physical activity in public health, Institute of Movement and Neurosciences, German Sport University Cologne, Cologne, Germany

**Keywords:** Paediatrics, Risk factors, Metabolic disorders, Translational research

## Abstract

A healthy lifestyle that includes physical activity has numerous positive effects on the mother and child during and after pregnancy. In this context physical activity plays a central role due to its influence on body composition. While visceral fatty tissue has a pro-inflammatory effect via so-called adipokines, myokines seem to have a more anti-inflammatory effect and thus prevent numerous diseases such as gestational hypertension or gestational diabetes. However, many women show a decreased level of physical activity during pregnancy when compared to pre-gestation levels. The reasons underlying this change are manifold and include concern about the effects of physical exertion on the unborn child. Gynaecologists and midwives are also often uncertain about what specific advice to give regarding physical activity. The present review describes, besides the underlying mechanisms, current physical activity recommendations and corresponding evidence with a focus on weight development in terms of obesity, gestational diabetes and foetal outcome.

## Background

The health benefits of physical activity for women during and after pregnancy are well documented [1–3]. Lifestyle interventions (diet and exercise) lead to a lower risk of developing gestational diabetes mellitus (GDM) [4] and excessive weight gain. Furthermore, postnatal persistence of weight gain occurs less frequently [5]. In a meta-analysis of 7278 patients from 44 studies, it has been shown that lifestyle interventions reduce risk of pre-eclampsia, pregnancy hypertension, premature birth and intrauterine death [3]. Despite these positive effects, many women exercise insufficiently even before pregnancy and reduce their activity further during pregnancy. In a non-pregnant population, current studies have shown that in the European Union, only 26.2% of women and 35.7% of men meet the World Health Organization exercise recommendation to complete at least 150 min of moderate endurance activity per week [6]. Although the prevalence in Germany is above the European Union average, with 42.6% for women and 48.0% for men, more than half of the German population invests too little time in physical activity [7]. This trend can also be observed in pregnant women [8]. In a study by Evenson et al. [9], it was show that only 15.8% of 1979 pregnant women who took part in the survey achieved the American physical activity recommendations of 30 min of moderate physical activity 3–5 times/week. Comparable results were also shown in studies from Spain (20.3%) [10] and the United States (16.0%) [11]. A recent survey of 83 pregnant German women showed that 41.0% reduced their physical activity during pregnancy, and 38.6% were already inactive before pregnancy and did not change their activity levels once pregnant. Only 9.6% of women increased their activity during pregnancy [12]. These results are supported by another German pilot study with 67 pregnant women [13]. Furthermore, this study noted that only 5.3% of overweight women and none of the obese women reached the physical activity recommendations [13].

The decreased physical activity level of pregnant women is partially due to pregnancy-associated side effects, such as fatigue, nausea or back pain as well as general weight gain [14–16]. Other reasons include concern about possible damage to the unborn child as well as miscarriage, premature birth or accidents during sports [8, 17] and lack of information. Surveys also show that key players (i.e., gynaecologists and midwives) are often inconsistent in their advice on physical activity [18]. It is known that women who feel well informed about physical activity by their gynaecologist continue their physical activity more often than pregnant women who did not feel well informed [12].

Therefore, in addition to the underlying mechanisms, this review will examine current physical activity recommendations and corresponding evidence with a focus on weight development in terms of obesity, GDM and foetal outcome.

## Definition of movement

Physical activity is generally described as any kind of movement that is accompanied by an increase in energy consumption [19]. Sports, in turn, is defined as planned, structured, repetitive activity with the aim of improving or maintaining fitness. Fitness is linked not only to physical or cardiopulmonary performance, but also to muscle strength and therefore body composition and flexibility. The ‘dose’ is expressed as energy expenditure, whereas ‘intensity’ is the rate of energy consumption during selected activities and usually expressed as VO2max (or relative to individual body weight) or metabolic units (Table 1).

**Table 1:** Classification of physical (in)activity.

• Metabolic equivalents or METs = ratio of working metabolic rate to resting state.
• Corresponds to the multiplication factor by which the resting oxygen consumption of 3.5 ml O_2_/(kg body weight × min) under load is increased.
• Low activity ≤3 METs or <4 kcal/min or less than 75 W.
• Moderate activity = 3–6 METs or 4–7 kcal/min or 75–10 0W or 40–60% of VO_2_max.
• Vigorous activity ≥6 METs or >7 kcal/min or more than 100 W or greater than 60% VO_2_max.
• Activities below 1.5 METs are considered inactive or sedentary; however, in order to clearly distinguish these from everyday activities, it makes more sense to take sitting or lying time into account.

## The benefits of lifestyle interventions on mother and child in the context of weight development and GDM

### Excessive weight gain

A Cochrane review by Muktabhant et al. [20] with 65 randomised controlled trials (RCTs) was conducted to evaluate the effects of healthy diet, exercise or the combination of both on the prevention of excessive weight gain in pregnancy. The authors found that all three interventions reduced the risk of excessive gestational weight gain (GWG) by 20% on average (average risk ration (RR) 0.8, 95% confidence interval (CI) 0.73–0.87). More specifically, women who maintained a healthy diet, exercised more, or applied both interventions were more likely to experience low GWG than those in control groups (average RR 1.14, 95% CI 1.02–1.27). Muktabhant et al. [20] concluded that exercise in particular is an important aspect for controlling weight gain during pregnancy. These results have been confirmed in a study by Haakstad and Bo [5] which included 105 women who were inactive prior to the start of the study. They showed that pregnant women who regularly took part in the supervised exercise programme (2 times/week for 60 min each) for 12 weeks gained excessive weight significantly less frequently and reduced weight much faster after delivery when compared with controls.

By nature, exercise alone is not sufficient to have a decisive influence on weight gain. Nutrition seems to be superior to exercise in terms of reduced GWG increase [20, 21]. Craemer et al. [21] showed in a current meta-analysis, that compared with routine prenatal care, nutrition-only interventions reduced GWG significantly, whereas exercise-only and combined interventions trended toward GWG within Institute of Medicine (IOM) guidelines, but did not reach statistical significance. The authors conclude, that these findings may be led back to the inclusion of different exercise types. Some included theoretical advice about exercise, while others performed intervention programmes, e.g. dancing vs. recommending a certain number of steps per day. In contrast, dietary recommendations do not show such diversity and focus mainly on the advice to eat more fruit and vegetables and/or to reduce the consumption of high sugar and/or high fat foods.

Unfortunately, there are no clear results in the target group of overweight and obese pregnant women. Many women in RCTs exceed weight gain recommendations despite participation in lifestyle interventions [22–24]. This may be due to insufficient participation in the programme or to the nature of the programmes [25], which can become quite challenging and result in feelings of discouragement, especially for overweight and obese pregnant women. Nevertheless, three publications [26–28] show the positive effects of lifestyle interventions on excessive weight gain in overweight and obese pregnant women.

The Lifestyle in Pregnancy [26] study offered dietary guidance, free membership at a fitness centre and personal coaching. Obese women showed a reduced mean GWG compared to the control group (7.0 vs. 8.6 kg, *p* = 0.01); however, there were no differences in excessive GWG (35.4% vs. 46.6%, *p* = 0.06) [26]. The Treatment of Obese Pregnant Women [27] study randomised obese women (*n* = 425) into a physical activity intervention with or without a dietary intervention. The physical activity intervention included encouragement to increase physical activity, aiming at a daily step count of 11,000 monitored by a pedometer. Dietary intervention included follow-up on a hypocaloric Mediterranean-style diet. Results showed that median values of GWG were lower in each of the intervention groups. Moreover, it was found that the physical activity intervention decreased GWG by a mean of 1.38 kg (*p* = 0.04) in a multivariate analysis, and women in the intervention group were more likely to meet GWG goals (49–55% intervention vs. 37% control, *p* = 0.01) [27].

In the longitudinal interventional study by Bogaerts et al. [28], obese pregnant women were randomised into three different groups: a control group, a brochure group receiving written information on maintaining a healthy lifestyle and an experimental group receiving an additional four antenatal lifestyle intervention sessions by a midwife trained in motivational lifestyle intervention. The analysis of variance showed a significant difference between the mean GWG of the control group of obese pregnant women (13.5 ± 7.3 kg), the brochure group (9.5 ± 6.8 kg) and the lifestyle intervention group (10.6 ± 7 kg).

Although there are some studies suggesting that excessive weight gain can be reduced by lifestyle interventions, the question generally arises as to how much weight, especially obese, women should gain or whether at least weight stagnation should not be aimed at. In most studies weight gain is defined according to the IOM criteria [29]. However, GWG guidelines are often not stratified by severity of obesity. Faucher and Barger [30] conducted a systematic review of original research and conclude that research suggests the lowest combined risk of selected outcomes (e.g. small-for-gestational-age, large-for-gestational-age babies) with weight gain of 5–9 kg in women with class I obesity, 1 kg to less than 5 kg for class II obesity and no GWG for women with class III obesity [30]. Kiel et al. [31] also confirm that limited or no weight gain in obese pregnant women has favourable pregnancy outcomes. However, observational studies are inconsistent regarding an optimal GWG range for obese women [32]. In a recent study, Thompson and Thompson [32] showed that weight gain of <5 kg in pregnancy by women with Class III obesity significantly increases the risk of low birth weight infants and neonatal mortality, relative to those gaining weight within the IOM limits. Therefore, more evidence is needed from RCTs, stratifying by obesity class, to define the amount of weight gain or stabilisation compatible with optimal outcomes for women with increasing obesity.

### Gestational diabetes mellitus (GDM)

Overall, animal studies have shown that exercise improves insulin sensitivity, lipid metabolism and glucose tolerance in diabetic and obese rodent pregnancies [33]. A study by Musial et al. [34] used a mouse model and aimed to determine the effects of obesity during pregnancy with and without an exercise intervention on maternal body weight and composition, nutrient handling, and insulin and lipid signalling in liver, skeletal muscle, and adipose tissue. It was found that exercise induced changes in the insulin and lipid signalling pathways in obese dams that differed from those observed in control and sedentary‐obese dams.

A similar outcome can also be observed in humans. Regular physical activity leads to a reduction in blood glucose concentration in both the fasting and postprandial states [35]. Pregnant women who require insulin cannot always prevent the use of insulin through exercise; however, a systematic review by Davenport et al. [36] showed that the required insulin dose can be reduced. Conversely, no higher incidence of possible hypoglycaemia has been found [36].

Regarding lifestyle interventions, previous Cochrane reviews which assessed dietary advice alone [37] and exercise interventions alone [38] have revealed inconclusive findings. In an updated version by Shepherd et al. [4], with 23 RCTs (involving 8918 women and 8709 infants) that compared combined diet and exercise interventions with no intervention (standard care), the authors found a possible reduced risk of GDM in the diet and exercise intervention group compared with the standard care group (RR 0.85, 95% CI 0.71–1.01) [4]. These results were confirmed in a current Cochrane review by Griffith et al. [39].

In considering lifestyle interventions for overweight and/or obese women, it becomes evident that no clear effects have been found (summarised in [40]). In a recent meta-analysis and meta-regression by Guo et al. [41], however, four core elements for successful prevention of GDM could be shown: targeting the high-risk population; an early initiation of the intervention; the correct intensity and frequency of exercise; and GWG management. Guo et al. [41] concluded that interventions are most effective in high-incidence populations rather than in individual women who are overweight or obese. Furthermore, exercise of moderate intensity for 50–60 min twice a week could lead to an ~24% reduction in GDM. In combination with nutrition several studies found positive effects on the incidence or risk of GDM [42, 43], although other studies found no effect [44, 45]. In an RCT by Koivusalo et al. [42], 293 women with a history of GDM and/or a pre-pregnancy BMI of ≥30 kg/m^2^ were enrolled in the study. The intervention group received individualised counselling on diet, physical activity, and weight control, and had one group meeting with a dietitian. The control group received standard antenatal care. The authors could demonstrate a reduction in the incidence of GDM by 39% in high-risk pregnant women as a result of the moderate individualised lifestyle intervention [42]. A prospective, RCT by Petrella et al. [43] could also demonstrate positive effects on the risk of developing GDM in overweight or obese women. Women were randomised to no intervention (*n* = 28) or a Therapeutic Lifestyle Changes Program (*n* = 33) including diet (overweight: 1700 kcal/day, obese:1800 kcal/day) and mild physical activity (30 min/day, 3 times/week). It was found that the intervention was an independent factor for preventing GDM (*R*^2^ = 0.15; *p* = 0.014) after adjusting for BMI > 30 kg/m^2^ (*p* = 0.38), age >35 years (*p* = 0.36), Caucasian ethnicity (*p* = 0.58) and the lack of family history of diabetes (*p* = 0.63) [43].

### Foetal outcome

The Cochrane review by Muktabhant et al. [20] also analysed the effects of lifestyle interventions on foetal outcome. Regarding macrosomia, the largest effect size was found in the supervised exercise-only intervention group (RR 0.81, 95% CI 0.64–1.02). Moreover, subgroup analysis by risk revealed that high-risk women (overweight or obese women, or women with or at risk of GDM) receiving combined diet and exercise counselling interventions experienced a 15% reduced risk of infant macrosomia [20]. A more recent publication by Barakat et al. [46] analysed the effects of supervised exercise on macrosomia. The RCT included an exercise group (supervised aerobic and strength training 3 times/week for 50 min each; *n* = 382) compared to a standard care control group (*n* = 383) and found a significant reduction in the number of infants born with macrosomia in the exercise group versus the control [46]. The incidence of macrosomia was only 1.8% (*n* = 7) in the exercise group compared to 4.7% (*n* = 18) in the control group. A meta-analysis by Wiebe et al. [47] that included 28 RCTs also reported a similar trend as the odds of having a large for gestational age (LGA) infant was reduced by 31% with prenatal exercise (odds ratio [OR] 0.69, 95% CI 0.55–0.86). Furthermore, the decrease in LGA infants did not increase the risk of small for gestational age (SGA) infants (OR 1.02, 95% CI 0.72–1.46).

There are few studies that have investigated the effect of combined diet and exercise intervention on overweight and obese women in terms of foetal parameters (LGA, SGA). Poston et al. [45] investigated the effect of a complex intervention addressing diet and physical activity on the incidence of LGA infants in 1555 obese pregnant women. The authors did not find any significant differences between intervention and control group regarding the incidence of LGA infants (RR 1.15, 95% Cl 0.83–1.59; *p* = 0.40). These findings are in line with results from Dodd et al. [48] who determined the effect of antenatal dietary and lifestyle interventions on health outcomes in 2212 overweight and obese pregnant women. The risk of the infant being LGA was not significantly different between the intervention group (lifestyle advice; incidence 19%) versus standard care (incidence 21%; adjusted RR 0.90, 95% CI 0.77–1.07; *p* = 0.24). However, infants born to women after lifestyle advice were significantly less likely to have a birth weight above 4000 g. In a recent post hoc analysis of an open-label RCT that included 82 women with a BMI ≥ 25 kg/m^2^, positive effects of a lifestyle intervention were found on perinatal outcome [49]. Women in the intervention group received a low glycemic index diet and were encouraged to spend 30 min/day walking at least 4 times/week. Women in the intervention group had a lower rate of LGA infants. Furthermore, the intervention did not increase the rate of SGA babies in the lifestyle intervention group [49].

## Postpartum weight retention

Several studies have examined the relationship between postpartum physical activity/lifestyle parameters and postpartum weight retention [50, 51]. A study by Ng et al. [51] with a prospective cohort of 2231 Australian women reported that women who consumed more than three serves of fruit/vegetables per day, engaged in recreational activity with their baby, and spend more time on leisure walking or with friends were at reduced risk for high postpartum weight retention. Oken et al. [50] examined the associations of postpartum television viewing, walking, and trans fat intake with weight retention equal to or greater than 5 kg at 12 months postpartum in 908 women. Women who watched less than 2 h of television, walked at least 30 min, and consumed trans fat below the median had an odds ratio of 0.23 (95% CI: 0.08–0.66) of retaining at least 5 kg [50].

With regard to the target group of overweight and obese women O’Toole et al. [52] examined the impact of an individualised, structured diet and physical activity intervention compared to self-directed intervention on weight loss in 40 overweight women during the first year postpartum. Women in the individualised, structured diet and physical activity group had a significant weight loss (−7.3 kg, *p* < 0.01), a significant decrease in percent body fat (−6%, *p* < 0.01), and no change in fat-free mass compared to self-directed group. The authors conclude that women who committed to an individualised, structured diet and physical activity programme had a high likelihood of successful weight loss that persisted at 1 year [52]. These findings are in line with current practice recommendations for postpartum obesity. The FIGO guidelines [53] recommend, that obese women should be offered further dietary and physical activity advice to support postpartum weight management. There is evidence that especially the combination of a healthy diet and physical activity seems to be effective in postpartum weight retention [54].

Additionally, growing evidence suggests that interpregnancy weight change is a risk factor for perinatal outcomes, since it may increase the probability of gestational complications including gestational diabetes. Therefore, FIGO guidelines strongly recommend that women should be informed that weight loss between pregnancies reduces the risk of stillbirth, hypertensive complications and fetal macrosomia in subsequent pregnancies [53].

## Select underlying mechanisms

The positive effects described above can be explained by a change in body composition. Visceral fatty tissue plays an important role in this context. It is an endocrine active organ and produces more than 600 so-called adipokines, which contribute to the regulation of metabolic processes such as insulin secretion, appetite/saturation, energy balancing as well as inflammation [55]. At a certain level and with permanent overfeeding, there is excessive secretion of these adipokines or a changed pattern of permanent low-grade inflammation and dysfunction of adipose tissue with increased levels of leptin, interleukin (IL-) 6, TNF-α, increased oxidative stress and a reduction of adiponectin [56] (Table 2).

**Table 2:** Adjustments in peripheral tissue in pregnant women; mod. according to [91] and [92].

	White fat tissue	Skeletal muscle	Liver	Possible consequences
Normal pregnancy	↓Insulin sensitivity ↑Expansion ↑Leptin ↓Adiponectin ↑Triglycerides ↑Lipolysis ↑IL-6	↑Oxidative stress ↑Lipid oxidation ↓Insulin sensitivity ↑Endoplasmic reticulum stress	↑Gluconeogenesis ↑Lipid oxidation ↓Insulin sensitivity	—Mild insulin resistance
Obesity/GDM	↑Inflammatory cytokines ↑Macrophage infiltration ↑↑Lipolysis ↓↓Insulin sensitivity ↑↑IL-6	↓↓Insulin sensitivity ↓Calcium signalisation ↑Endoplasmic reticulum stress ↑Oxidative stress ↓Antioxidant capacity/defence ↓Lipid oxidation	↓↓Insulin sensitivity ↑ Fat accumulation	—Hyperglycaemia —Severe insulin resistance —Hyperlipidaemia —Leptin resistance

*GDM* gestational diabetes mellitus, *IL-6* Interleukin 6.

In contrast, physical activity acts as a kind of protective factor against the diseases described above [57], while physical inactivity supports the development of inflammation [58]. The reason for this likely involves muscle mass. Skeletal muscle constitutes the largest organ of the body, and its energy production and consumption are fundamental for controlling metabolism. Skeletal muscle is also considered an endocrine organ that produces hundreds of myokines, such as IL-4, IL-6, IL-7 and IL-15, myostatin, myonectin, follistatin-like 1, leukaemia inhibitory factor and irisin [59, 60]. These myokines not only act locally in the muscle in an autocrine/paracrine manner, but are also released into the bloodstream via muscle contraction as endocrine factors to regulate physiological processes in other tissues. It is generally understood that the various health-promoting effects associated with physical activity can help prevent minor inflammatory diseases such as type 2 diabetes, insulin resistance or metabolic syndrome.

In the context of pregnancy, Van Poppel et al. [61] showed in their study of 46 overweight pregnant women that increased physical activity was associated with significantly higher IL-6 at all measured timepoints (15th, 24th, and 32nd week of gestation) and with higher TNF-α in the 15th week of gestation. The authors suggest that higher levels of IL-6 in more active women represent muscle-derived IL-6, which is anti-inflammatory and associated with increased lipolysis and fat oxidation as well as inhibition of TNF-α, which in turn is associated with improved insulin sensitivity [62]. Further research is needed to elucidate the mechanisms underlying the effects of physical activity during pregnancy.

## Physical activity recommendations in general and in the context of obesity and/or gestational diabetes

As clear as the benefits of exercise during pregnancy are, the recommendations for exercise provided by healthcare professionals and societies are inconsistent. Before starting with an exercise programme, a medical examination is recommended to exclude possible risks (see Tables 3–5) [63]. Based on this, all pregnant women without contraindications are recommended to exercise at least 150 min per week [64]. Due to the fact that there are hardly any concrete studies on selected forms of exercise during pregnancy, the focus of the recommendations is generally on endurance and strength training.

**Table 3:** Absolute contraindications [mod. according to [83]].

• Hemodynamically relevant heart disease
• Restrictive lung disease
• Incompetent cervix or cerclage
• Premature labour during the current pregnancy or multiple gestation at risk of premature labour
• Persistent second- or third-trimester bleeding
• Placenta praevia after 26 weeks of gestation
• Ruptured membranes
• Pre-eclampsia or pregnancy-induced hypertension
• Severe anaemia

**Table 4:** Relative contraindications [mod. according to [83]].

• Anaemia
• Unevaluated maternal cardiac arrhythmia
• Chronic bronchitis
• Poorly controlled diabetes mellitus type 1
• Extreme morbid obesity
• Extreme underweight (BMI < 12 kg/m^2^)
• History of extremely sedentary lifestyle
• Intrauterine growth restriction during the current pregnancy
• Poorly controlled hypertension
• Orthopaedic limitations
• Poorly controlled seizure disorder
• Poorly controlled hyperthyroidism
• Severe nicotine abuse

*BMI* body mass index.

**Table 5:** Warning signals [mod. according to [63, 83]].

• Vaginal bleeding
• Abdominal pain
• Regular painful contractions
• Loss of amniotic fluid
• Dyspnoea before exertion
• Dizziness
• Headache
• Chest pain
• Muscular weaknesses that affect balance
• Lower leg/calf pain or swelling

## Type of exercise

Everyday activities and unstructured activities: Unstructured activities refer to types of activities that are in the light to moderate intensity range and usually part of daily living (e.g., cycling, climbing stairs and walking). Unstructured activity forms the basis for physical activity recommendations. In general, a daily number of 10,000 steps per day is recommended [65]. Walking/Nordic walking is very popular among pregnant women and can be done anywhere. Pedometers or corresponding apps contribute significantly to motivation and can be used as forms of support [66, 67].

Sports and structured activities: structured activities constitute types of activities that range from a moderate to vigorous intensity. They should be done regularly in addition to everyday activities. A basic distinction is made between weight-bearing and non-weight-bearing exercises, which have different effects on the body [68, 69]. Weight-bearing exercises are more energy-intensive, as a person’s own body weight is carried during the exercises; however, they are usually well tolerated by previously active pregnant women. Non-weight-bearing exercises are activities in which the body weight is not carried, such as swimming.

At the centre of the physical activity recommendations are, after warming up or cooling down, generally low-impact loads as well as aerobic endurance loads, strength training for the large muscle groups, and non-weight-bearing exercises without great burdens on the joints. These include, for example, swimming and cycling/cycling ergometer [56, 65, 70]. Swimming and water gymnastics are particularly beneficial non-weight-bearing activities/exercises, as peripheral oedema is reduced, joint stress is minimised and loss of balance and falls are of little concern [71]. Two large cohort studies from Denmark and England [72, 73] investigated the effects of swimming on birth weight and foetal outcome and found no association between swimming duration and birth weight. Swimming is considered safe and effective according to the authors [72–74].

Walking and Nordic Walking are also described as suitable activities [63, 75]. Regarding moderate strength training, there are limited study findings available. In summary, it can be stated that light intensity resistance training with low weights lifted through a dynamic range of motion in multiple repetitions is safe and effective during pregnancy [76–78].

In addition to endurance and strength training, pregnancy-specific yoga, Pilates and gymnastics are recommended;[79] however, care must be taken to avoid exercises involving the supine position or prolonged standing. Studies have shown that these positions reduce venous return and cause hypotension in 10–20% of pregnant women [80–82].

Finally, setback games (e.g., tennis and table tennis) as well as jogging can still be carried out during pregnancy when considering prior experience and the training condition. Due to the physiological hormonal changes throughout pregnancy, the stability of the joints is increasingly reduced, which may lead to a higher susceptibility to injuries during rapid start-stop movements. Fast and/or jerky movements should therefore be avoided in the activities mentioned [63, 75, 83].

## Duration, frequency and intensity

In terms of structured activities, it is generally recommended to exercise 150 min per week. According to the American College of Obstetricians and Gynecologists (ACOG) [63], an exercise programme that leads to an eventual goal of moderate-intensity activity for at least 20–30 min per day on most or all days of the week should be developed with the patient and adjusted as medically indicated. Mottola and Artal [56] support these findings and recommend training at least 3 times/week for at least 25 min at mild or moderate intensities but not more than 40 min at higher intensities.

Depending on the country of origin, there are slightly different recommendations for the intensity of physical activity. In terms of absolute intensity, some countries specify age-related heart rate zones [79, 84]. Target heart rate zones provided by Canada represented 60–80% of maximum aerobic capacity [75, 79]. This is also recommended in the current ACOG guidelines [63]. In contrast, the British guideline recommends an upper range of 60–90% of maximum heart rate for women who want to maintain their fitness during pregnancy [84] and 60–70% of maximum heart rate for women who were rather inactive before pregnancy. Mottola and Artal [56] recommend training at 30% of one’s heart rate reserve (HRR; mild) or a 70% HRR (moderate) intensity.

Almost all international recommendations include the relative intensity in their recommendations for action [75]. Thus, it is recommended to use subjective stress perception as a marker for the intensity [63, 75]. The exercise should be ‘*somewhat strenuous’* (on the 6–20 Borg Scale at 13–14). Use of the talk-test as an additional method to determine moderate intensity is also recommended; pregnant women should be able to talk to a partner while practicing the activity [63, 75].

Absolute and relative contraindications for which physical activity is only recommended to a limited extent or not at all are listed in Tables 3 and 4. Certainly, some of these contraindications reflect the caution of the gynaecologists and obstetricians in charge, i.e., in the context of morbid obesity or a pronounced sedentary lifestyle, physical activity adapted to the individual condition is indicated but listed under relative contraindications. The question becomes ‘what kind of exercise can be recommended, and to whom?’ Even if there are possible risks, inactivity does not necessarily have to be advised, although the form of exercise may have to be adapted accordingly.

With the following warning signals (Table 5), the sport must be stopped and a medical check-up must take place first.

### Obesity/GDM

**Fig. 1:**
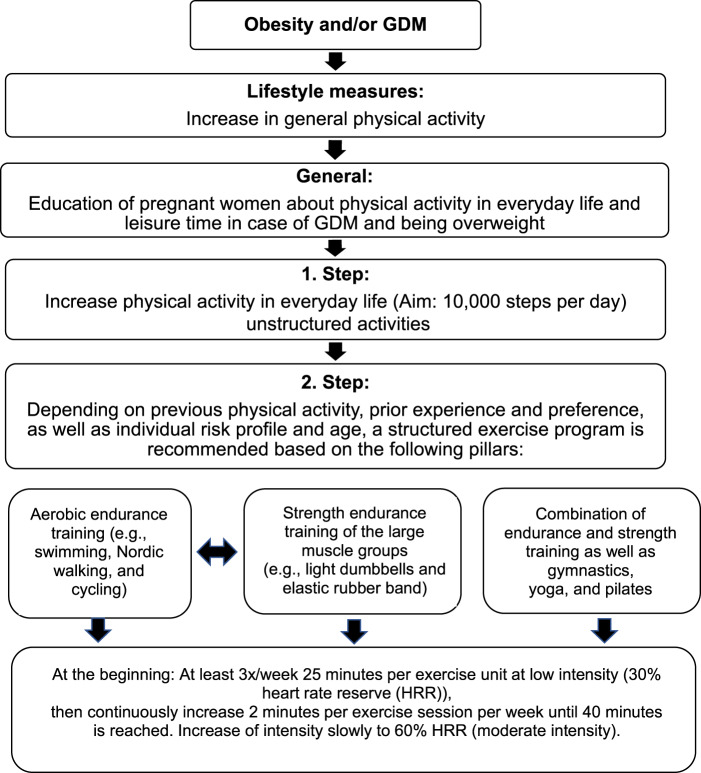
Graphical display of physical activity recommendations, mod. according to [90]. Recommended types of exercise, the duration, frequency and recommended intensity especially related to women with obesity and/or gestational diabetes mellitus (GDM). It starts with general recommendations that can be gradually differentiated.

In the context of obesity, the same information on the type of exercise applies as previously described; however, slightly modified recommendations are given regarding duration, frequency and intensity. It is recommended to start with 25 min per exercise session at a low intensity (30% HRR) corresponding to 30% of the maximum minus the resting HRR at least three times per week, then continuously increase by 2 min per exercise session per week until 40 min is reached [56, 65]. The intensity should be slowly increased up to 60% HRR (moderate intensity) (see Fig. 1). With regard to morbid obesity/obesity class III (BMI ≥ 40 kg/m^2^), there are no recommendations on physical activity during pregnancy. However, it seems advisable to focus on everyday activities and gradually increase them, as recommended for overweight/obese pregnant women. Moreover, individual needs and capabilities should be taken into consideration [53]. A qualitative study by Denison et al. [85] has shown that pregnant women, especially those who are morbidly obese, received no or inadequate information, advice and support regarding physical activity in pregnancy. As a result, women were uncertain as to what they could and could not do in pregnancy, and of the benefits and risks of physical activity in pregnancy. Walking is a very popular activity during pregnancy. Considering previous experience, health status and capabilities, the daily number of steps can be documented and slowly increased by means of a pedometer, for example. Pedometers were viewed as a good motivational support to increase physical activity levels in pregnancy [85]. Beside walking, swimming was the most frequently cited activity type, although barriers to swimming were reported by some women.

The abovementioned recommendations also apply in the context of GDM. Physical activity is best performed 30 min postprandial every day, but at least 3 days a week [64, 86]. It is also recommended to start with 30% HRR or a target exercise energy expenditure of ≥16 MET-h per week or gradually increasing the % HRR to 60% with a target of 28 MET-h per week [56]. The goal here is also to achieve 40 min per exercise session. A combination of endurance and strength training is recommended for all pregnant women. If blood glucose levels are regularly elevated at the same time of day, most often after breakfast, it may be recommended that physical activity—if possible—be executed during this time. It can be very motivating if the women themselves can see the positive effect of exercise based on blood sugar measurements; however, intensive exercise can also lead to an increase in blood glucose levels. To avoid misconceptions, this should be addressed by healthcare professionals.

## Conclusion

A healthy lifestyle, especially one which incorporates exercise and nutrition, is essential in the prevention of excessive weight gain or the development of GDM and its treatment. In principle, pregnant women in this phase of life seem to be very receptive to advice and information, as they are concerned about the well-being of the child and are keen to make positive changes. However, reality shows that most pregnant women do not engage in enough exercise [9–11]. Apart from physical reasons (e.g., back pain and water retention), concerns about possible damage to the unborn child, a miscarriage or premature birth, or accidents during sports [8, 17] are often cited in the reduction of physical activity during pregnancy. Conversely, there are also some women who would like to continue their sports activities but express frustration due to a lack of specific guidance or education about the appropriate types, intensity, duration, or frequency of exercise that would be safe for their pregnancy [13, 87]. Anxiety and frustration might lead to inactivity during pregnancy and its negative consequences, such as excessive maternal weight gain and high postpartum weight retention.

Medical doctors therefore play an important role in the consultation. Studies showed that maternal weight is better controlled, physical activity is increased and more attention is paid to a balanced and healthy nutrition if the physician provides advice in these areas [88]. It should be noted that physicians are also uncertain about concrete recommendations concerning physical activity during pregnancy. A study by McGee et al. [18] showed that all gynaecologists who took part in the survey provided appropriate advice on aerobic exercise, but their advice on resistance training, intensity of exercise and third-trimester exercise were often discordant with ACOG’s guidelines [18]. Doctors should therefore be trained in current physical activity recommendations. However, there are hardly any recommendations for morbid obese pregnant women regarding physical activity. Moreover, it remains unclear as to how much weight, especially morbid obese, women should gain or whether at least weight stagnation should not be aimed at. Although there is a lack of information about concrete recommendations doctors should be made more aware of the topic of healthy lifestyles.

The learning and use of special techniques such as motivational counselling can support the success of counselling. According to Lindhardt et al. [89], time must be dedicated to the technique, but as a result, it enables the healthcare professionals to become more proficient and assists them in handling difficult workloads. By using this technique, for example, possible fears and barriers to exercise during pregnancy can be overcome, and pregnant women can be motivated to adopt a healthy and active lifestyle. For instance, the current ACOG guidelines [63] also recommend that all gynaecologists, midwives and other obstetric caregivers use this strategy and the five A’s (Ask, Advise, Assess, Assist, and Arrange). The use of motivational counselling or interviewing has also been studied in overweight pregnant women. Lindhardt et al. [89] showed that motivational interviewing is a useful method when communicating with obese pregnant women, as this target group in particular is considered to be difficult to motivate. As such, there is a need for further translational studies on the short- and long-term effects of physical activity during pregnancy to elucidate the mechanisms underlying the effects of exercise as well as the concrete recommendations of healthcare professionals. This is an important step towards public health and the early prevention of non-communicable diseases.
